# Risk Factors for Infection With Multidrug-Resistant Gram-Negative Bacteria in a Tertiary Care Hospital in Saudi Arabia: A Case-Control Study

**DOI:** 10.7759/cureus.37291

**Published:** 2023-04-08

**Authors:** Abdulhakeem Althaqafi, Muhammad Yaseen, Fayssal Farahat, Adeeb Munshi, Abdulfattah Al-Amri, Sabiha Y Essack

**Affiliations:** 1 College of Medicine, King Saud Bin Abdulaziz University for Health Sciences, Jeddah, SAU; 2 Infectious Diseases, King Abdullah International Medical Research Center, Jeddah, SAU; 3 Medicine/Infectious Diseases, King Abdulaziz Medical City, Jeddah, SAU; 4 Infection Prevention and Control, Bradford Teaching Hospital National Health Service (NHS) Foundation Trust, Bradford, GBR; 5 Public Health and Community Medicine, King Saud Bin Abdulaziz University for Health Sciences, Riyadh, SAU; 6 Public Health and Community Medicine, Menoufia University, Menoufia, EGY; 7 Infection Prevention and Control, King Abdulaziz Medical City Riyadh, Riyadh, SAU; 8 Microbiology, King Abdullah International Medical Research Center, Jeddah, SAU; 9 Microbiology, King Abdulaziz Medical City, Jeddah, SAU; 10 Antimicrobial Research Unit, University of Kwazulu-Natal, Durban, ZAF

**Keywords:** saudi arabia, colonized, infected, risk factors, multidrug resistance, gram-negative

## Abstract

Background

The increase in the incidence of multidrug-resistant (MDR) organisms especially Gram-negative bacteria (GNB) in healthcare facilities is a serious cause of concern. This study identified risk factors for the infection with these MDR GNB, such as *Acinetobacter baumannii, Klebsiella pneumoniae, Pseudomonas aeruginosa*, and *Escherichia coli* to inform healthcare workers about strategies for their containment.

Methods

A case-control study was carried out at a tertiary care hospital where 100 patients with healthcare-associated infections (infections arising 48 hours after admission) caused by MDR GNB were compared with two control groups, i.e., 100 patients with healthcare-associated infections caused by non-MDR GNB (not meeting the criteria of MDR) and 100 patients without infection caused by GNB. MDR bacteria were defined as the ones that were non-susceptible to at least one antibiotic in three or more classes of antibiotics. The data were analyzed using descriptive statistics (frequency and percentage of categorical variables). Multivariate regression analysis was undertaken to identify significant predictors of MDR GNB. Odds ratios with 95% confidence intervals were calculated, and the level of significance was determined at p-value < 0.05.

Results

A total of 388 organisms were isolated during four months (January-April 2015) from 332 patients. Fifty-six (17%) of the patients were infected with more than one organism. Among the MDR bacteria, the most dominant MDR organism was *A. baumannii* (38%), followed by *K. pneumoniae* (31%), *P. aeruginosa* (20%), and *E. coli* (11%). Among the non-MDR organisms, the most dominant was* P. aeruginosa* (47%), followed by* E. coli (32%), K. pneumoniae* (18%), and *A. baumannii* (3%).

Patients with MDR organisms compared with the first control group (patients with non-MDR organisms) showed that prior antibiotic use (p-value: 0.001), intensive care unit (ICU) admission (p-value: 0.001), and indwelling medical devices (p-value: 0.005) were significant risk factors for MDR infections. It was also found that the risk factors for MDR GNB infection were the same in the second control group (patients without infection): prior antibiotic use (p-value: 0.002), ICU admission (p-value: 0.001), and indwelling medical devices (p-value: 0.03). Based on the comparison of the two control groups, prolonged hospital stays of more than five days (p-value: 0.001), immunosuppressive therapy (p-value: 0.02), and over 60 years of age (p-value: 0.02) were significant risk factors for non-MDR infection.

Conclusion

The risk factors identified in our study provide guidance to healthcare workers for the prevention and containment of MDR GNB.

## Introduction

Increase in the incidences of multidrug-resistant (MDR) bacteria in healthcare facilities is a significant concern [1]. The World Health Organization (WHO) has published a priority pathogens list (PPL) of antibiotic-resistant bacteria to guide the research and development of new antibiotics. The priority pathogens have been categorized into three major priorities, i.e., critical, high, and medium. The critical priority pathogens include common Gram-negative bacteria (GNB) like carbapenem-resistant *Acinetobacter baumannii (A. baumannii)*, *carbapenem-resistant Pseudomonas aeruginosa (P. aeruginosa)*, carbapenem-resistant and third generation cephalosporin-resistant Enterobacteriaceae like *Klebsiella pneumoniae (K. pneumoniae)* and *Escherichia coli (E. coli)* [2].

Several studies conducted worldwide have shown that GNB have overtaken their Gram-positive counterparts as far as the resistance to common antimicrobials is concerned [3-5]. GNB have a variety of mechanisms to develop resistance to currently used antibiotics. They can rapidly transfer these mechanisms to other bacteria through various mobile genetic elements [6]. Several studies in Saudi Arabia have also found that MDR GNB have become more prevalent than Gram-positive ones, especially *A. baumannii, P. aeruginosa, K. pneumoniae and E. coli*. [7-11].

Identification of risk factors for MDR GNB has important implications for patient care. Several risk factors have been associated with acquiring antibiotic-resistant pathogens in the hospital. Widespread and inappropriate use of antibiotics in the hospital, intensive care unit (ICU) stay, presence of invasive devices, surgical procedures, and severe co-morbid conditions are commonly reported risk factors for infection with MDR GNB. Other risk factors include age >65 years, duration of hospitalization, surgery, wounds (surgical or traumatic), previous antibiotic therapy, especially within 90 days, current antibiotic therapy, mechanical ventilation, urinary catheterization, nasogastric intubation, central venous and peripheral catheters, prior hospitalization and transfer from another unit or hospital [12-14]. 

This article was previously presented as an abstract in “Patient Safety Forum 2019, Conference Proceedings, Kingdom of Saudi Arabia, Ministry of National Guard Health Affairs” in April 2019. Our study aimed to identify the risk factors for infection with MDR GNB to inform health care workers about the strategies for their containment.

## Materials and methods

Ethical considerations

The study was approved by the Institutional Review Board (IRB) of King Abdullah International Medical Research Center (KAIMRC) in Saudi Arabia (RJ13/032/J) and the Biomedical Research Ethics Committee (BREC) at the University of Kwazulu-Natal (BE279/13). All data were anonymously collected and interpreted. Additionally, all patients signed a general consent form during hospital admission as part of the hospital policy. 

Study design setting

The study was conducted from January to April 2015 at King Abdulaziz Medical City (KAMC), a tertiary care hospital in the city of Jeddah in the Western region of Saudi Arabia. KAMC is an over 500-bed tertiary care hospital serving the National Guards and their eligible dependents in the Western region of Saudi Arabia.

Bacterial isolates were collected prospectively from routine clinical samples from patients presenting with bloodstream, respiratory tract, skin, soft tissues, and urinary tract infections. The organisms included: *A. baumannii, K. pneumoniae, P. aeruginosa, and E. coli*. Only organisms with healthcare-associated infections were included in the study using the standard definition of 48 hours after admission [15]. Each organism was included in the study only once, at the time of the first positive result. MDR and non-MDR GNB were included (non-MDR were defined as the GNB that did not meet the definition criteria for MDR). The definition of the MDR by the experts from the European Centre for Disease Prevention and Control (ECDC) and the Centers for Disease Control and Prevention (CDC) was used for this study, where MDR were defined as the non-susceptibility to at least one antibiotic agent in three or more classes of antibiotics [16]. Isolates were categorized as MDRs or susceptible based on their minimum inhibitory concentrations (MICs) determined on the automated Vitek® 2 System (bioMérieux, USA) and interpreted according to Clinical and Laboratory Standards Institute (CLSI) guidelines [16].

A case-control design was used to study significant risk factors for the acquisition of MDR organisms by comparing cases (patients infected with MDR) with two control groups. All patients in the three groups were admitted during the four-month period from January to April 2015. The selection of the patients was based on the date of admission, microbiology culture results, and clinical judgment. Cases were matched with both control groups by type of ward (medical, surgical, or ICU) and hospital admission date. Each group consisted of 100 patients, and 300 patients were included in the study. The first control group included patients infected with non-MDR GNB. The second control group comprised patients without infection. 

For each patient, the following clinical data were collected prospectively in a checklist of different parts, including diagnosis at admission, type of clinical ward, having received antimicrobials in the past 90 days, previous hospitalization in the past 90 days, prolonged hospital stay (>= five days), ICU admission, immunosuppressed patients (patients on active malignancy or on chemotherapy, post organ transplant on immunosuppressive medications including steroids, and human immunodeficiency virus (HIV) with CD4 < 200), indwelling medical devices, recent surgery or trauma in the past 90 days, age >=60, gender and emergency admission.

Statistical analysis

Data were analyzed using IBM SPSS version 24.0 (IBM Corp., Armonk, NY). Descriptive statistics (frequency and percentage of categorical variables) were used. Multivariate regression analysis was undertaken to identify significant predictors of MDR-GNB. Variables included in multivariate regression analysis are receiving antimicrobials, previous hospitalization, prolonged hospital stay, ICU patients, immunosuppressed, indwelling medical devices, recent surgery or trauma, gender, and emergency admission. Odds ratios with 95% confidence intervals were calculated. The level of significance was determined at p-value < 0.05.

## Results

Between January and April 2015, 388 GNB mentioned above were isolated from routine clinical specimens sent for culture and sensitivity from patients admitted to the hospital. These specimens were collected from 332 patients. Fifty-six (17%) of the patients were infected with more than one organism. As mentioned earlier, only organisms with healthcare-associated infections were included in the study. The organisms included were: *A. baumannii* (80),* K. pneumoniae* (94), *P. aeruginosa* (129), and *E. coli *(85). These coincidently consisted of 194 MDR GNB and 194 non-MDR GNB. Among the MDR bacteria, the most dominant MDR organism was *A. baumannii* (38%), followed by *K. pneumoniae* (31%), *P. aeruginosa* (20%), and *E. coli* (11%). Among the non-MDR organisms, the most dominant was* P. aeruginosa* (47%), followed by* E. coli* (32%), *K. pneumoniae* (18%), and *A. baumannii* (3%).

Among the 100 cases (MDR patients), 51 were male, and 49 were female; 71 patients were admitted through the emergency department, and 29 were elective admissions. Among the 100 control patients (non-MDR patients), 58 were male, and 42 were females; 60 patients were admitted through the emergency department, and 40 were elective admissions. Among the 100 control patients (patients without infection), 53 were male, and 37 were females; 79 were admitted through the emergency department, and 21 were elective admissions. Among the 300 overall patients included in the case-control study, 145 (48%) were male, 155 (52%) were females, and 147 (49%) were above the age of 60 years. Two hundred and thirty-seven (79%) were admitted through the emergency department, and 63 (21%) were elective admissions. As far as the other risk factors are concerned, 186 (62%) received antimicrobial therapy within the last 90 days, 112 (37%) had previous hospitalization within 90 days, 200 (67%) had a prolonged hospital stay of more than five days, 70 (23%) were admitted in the ICU, 72 (24%) received immunosuppressive therapy, 166 (55%) had indwelling medical devices in situ, and 106 (35%) have had recent surgery or trauma (Table 1). 

**Table 1:** Risk factors for acquisition of multidrug-resistant (MDR) gram-negative bacteria (GNB) MDR: Multidrug-resistant; GNB: Gram-negative bacteria; OR: Odds Ratio; CI: Confidence Interval

Variables	Number (percentage)	Group			Multivariate regression		Multivariate regression		Multivariate regression	
					MDR GNB vs.non-MDR		MDR GNB vs. No infection		Non-MDR vs. No infection	
					MDR	Non-MDR	No infection	OR (95%CI)	P. value	OR (95%CI)	P. value	OR (95%CI)	P. value
		Receiving antimicrobial										
Yes	186 (62.0%)	84 (45.2%)	57 (30.6%)	45 (24.2%)	5.50 (2.19, 13.84)	0.001	3.98 (1.68, 9.44)	0.002	1.13 (0.53, 2.17)	0.72
No	114 (38.0%)	16 (14%)	43 (37.7%)	55 (48.2%)						
Previous hospitalization										
Yes	112 (37.3%)	44 (39.3%)	34 (30.4%)	34 (30.4%)	1.11 (0.51, 2.41)	0.79	1.05 (0.48, 2.33)	0.9	1.06 (0.53, 2.09)	0.88
No	188 (62.7%)	56 (29.8%)	66 (35.1%)	66 (35.1%)						
Prolonged hospital stay										
Yes	200 (66.7%)	77 (38.5%)	76 (38.0%)	47 (23.5%)	0.46 (0.19, 1.10)	0.08	1.95 (0.89, 4.26)	0.09	3.37 (1.74, 6.52)	0.001
No	100 (33.3%)	23 (23.0%)	24 (24.0%)	53 (53.0%)						
ICU patients										
Yes	70 (23.3%)	52 (74.3%)	11 (15.7%)	7 (10.0%)	11.11 (4.58, 26.93)	0.001	8.60 (3.28, 22.57)	0.001	1.43 (0.48, 4.29)	0.52
No	230 (76.7%)	48 (20.9%)	89 (38.7%)	93 (40.4%)						
Immunosuppressed										
Yes	72 (24.0%)	18 (25.0%)	33 (45.8%)	21 (29.2%)	0.55 (0.24, 1.27)	0.16	1.22 (0.48, 3.11)	0.68	2.43 (1.18, 5.04)	0.02
No	228 (76.0%)	82 (36.0%)	67 (29.4%)	79 (34.6%)						
Indwelling medical devices										
Yes	166 (55.3%)	79 (47.6%)	50 (30.1%)	37 (22.3%)	2.95 (1.39, 6.27)	0.005	2.43 (1.11, 5.33)	0.03	1.29 (0.67, 2.48)	0.45
No	134 (44.7%)	21 (15.7%)	50 (37.3%)	63 (47.0%)						
Recent surgery or trauma										
Yes	106 (35.3%)	42 (39.6%)	37 (34.9%)	27 (25.5%)	1.41 (0.68, 2.94)	0.36	1.60 (0.75, 3.39)	0.22	1.26 (0.63, 2.54)	0.51
No	194 (64.7%)	58 (29.9%)	63 (32.5%)	73 (37.6%)						
Age										
>60	147 (49%)	55 (50.0%)	55 (50.0%)	37 (40.2%)	0.79 (0.38, 1.66)	0.54	2.54 (1.14, 5.69)	0.02	2.12 (1.13, 3.99)	0.02
<=60	153 (51%)	45 (50.0%)	45 (50.0%)	63 (58.3%)						
Gender										
Male	145 (48.3%)	49 (50.0%)	49 (50.0%)	47 (49.0%)	1.24 (0.60. 2.54)	0.57	1.34 (0.64, 2.78)	0.44	1.34 (0.71, 2.51)	0.36
Female	155 (51.7%)	51 (50.0%)	51 (50.0%)	53 (51.0%)						
Emergency admission										
Yes	237 (79.0%)	71 (44.9%)	87 (55.1%)	79 (47.6%)	0.42 (0.17, 1.05)	0.06	0.45 (0.18, 1.13)	0.09	1.36 (0.57, 3.23)	0.49
No	63 (21.0%)	29 (69.0%)	13 (31.0%)	21 (61.8%)						

The comparison between patients with MDR organisms and the first control group (patients with non-MDR organisms) showed that previous antibiotic use (odds ratio {OR}: 5.50, 95% CI: 2.19, 13.84, p-value: 0.001), ICU admission (OR: 11.11, 95% CI: 4.58, 26.93, p-value: 0.001), and indwelling medical devices (OR: 3.02, 95% CI: 1.45, 6.33, p-value: 0.005) were the significant risk factors for the MDR infection (Table 1). The comparison between patients with MDR organisms and the second control group (patients without infection) also showed the same risk factors: previous antibiotic use (OR: 3.98, 95% CI: 1.68, 9.44, p-value: 0.002), ICU admission (OR: 8.60, 95% CI: 3.28, 22.57, p-value: 0.001), and indwelling medical devices (OR: 2.43, 95% CI: 1.11, 5.33, p-value: 0.03) as the significant risk factors for the MDR GNB infection. The comparison of the two control groups showed that prolonged hospital stay of more than five days (OR: 3.37, 95% CI: 1.74, 6.52, p-value: 0.001), receiving immuno-suppressive therapy (OR: 2.43, 95% CI: 1.18, 5.04, p-value: 0.02) and above 60 years age (OR: 2.12, 95% CI: 1.13, 3.99, p-value: 0.02) were the significant risk factors for infection by non-MDR organisms (Table 1).

## Discussion

The increasing incidence of MDR GNB is of great concern because of their negative impact on morbidity and mortality, exacerbated by the shortage of alternative therapeutic options [17,18]. Identifying patients at high risk of harboring common MDR GNB has important implications for patient care [12]. Our study identified previous antibiotic use, ICU admission, and indwelling medical devices as significant risk factors for the acquisition of MDR GNB, which is consistent with the previous studies [12,19].

The future use of the new WHO recommendations, and in particular, systematic screening based on well-identified risk factors, will enable earlier identification of colonized or infected patients to limit the spread of these microorganisms by taking adequate measures based on evidence-based practices [20].

Kalam et al. (2014 ) looked at the risk factors for carbapenem-resistant Gram-negative bacteremia. Only adult patients were included in the study. Their results revealed that age > 50 years, septic shock on presentation, ICU stay of > 72 hours, and receiving immunosuppressive medications were the most significant risk factors [21]. Another study was conducted by Mohsenpour et al. (2016) in Iran to identify the risk factors for imipenem-resistant GNB [22]. Their results showed that ICU stays, a history of antibiotic use, and central venous catheter insertion were the most significant risk factors associated with the acquisition of imipenem-resistant GNB [22]. Ting et al. investigated the risk factors for the acquisition of carbapenem-resistant (CR) GNB by comparing CR GNB-infected patients (Group 1) with patients who were infected with non-CR GNB (Group 2) [23]. They found out that previous exposure to antibiotics, especially carbapenem and anti-pseudomonal penicillin, and cephalosporin, together with a longer length of hospital stay, were the most significant risk factors for the development of CR GNB bacteremia [23]. 

In Saudi Arabia, a study was conducted by Al-Otaibi et al. (2016) in an oncology center to identify the significant risk factors associated with the bacteremia caused by the MDR GNB [24]. The study included both adult and pediatric patients. The bacteria included in the study were *E. coli* (29.5%), *A. baumannii *(18.0%), *Pseudomonas spp*. (16.3%) and *K. pneumonia *(13.1%). Other organisms included: *Salmonella spp. *(6.5%), *Serratia marcescens* (4.9%), *Enterobacter spp*. (3.24%). Results revealed that ICU admission (32.1%), surgical procedure (23.2%), and having a central line (21.4%) were the most significant risk factors [24]. Another study conducted in Saudi Arabia by Al Hamdan et al. (2022) revealed MDR GNB were most frequently identified in female patients (66.4%) and those aged 20-29 years (21.8%) [25]. The most frequently isolated GNB were *E. coli *(53.8%). Over the two years of the study, *Enterobacter* species had the highest rate of multidrug resistance (86.5%). A significant direct risk factor for the development of MDR GNB was mechanical ventilation for more than three days [25].

Our study found that the MDR GNB *A. baumannii* and *K. pneumoniae* were more dominant than *P. aeruginosa* and *E. coli.* Risk factors like previous antibiotic use, ICU admission, and indwelling medical devices were the significant risk factors in patients who acquired MDR GNB when compared with the other two groups of patients who were infected with non-MDR GNB or did not have an infection. Prolonged hospital stay, immunosuppression, and age were the most significant risk factors in the patients who were infected with non-MDR organisms compared with patients who had no infection. More effective and efficient antimicrobial stewardship programs, together with better infection control practices, especially in ICU, to prevent transmission, are recommended to address the problem. Implementing the WHO recommendations will undoubtedly add an extra dimension to prevent and control MDR GNB infections in healthcare settings [20]. The study has various limitations, including a single-center design and a small number of the patient. Another limitation of the study is that some risk factors were not analyzed, such as long-stay in nursing homes, patients with chronic ulcers, patients on hemodialysis, or patients with burns.

## Conclusions

The risk factors for MDR GNBs have not been well studied in Saudi Arabia. *A. baumannii* (38%), followed by *K. pneumoniae* (31%), were the most dominantly isolated organism among MDR bacteria. Our data also revealed that antibiotic therapy, long ICU stays, and indwelling medical devices were the most significant risk factors in patients infected with MDR GNBs. These data will stress the importance of continuous surveillance systems and antibiotic stewardship programs as strategies for reducing the number of MDR GNBs.
